# Topology-aware illumination design for volume rendering

**DOI:** 10.1186/s12859-016-1177-4

**Published:** 2016-08-19

**Authors:** Jianlong Zhou, Xiuying Wang, Hui Cui, Peng Gong, Xianglin Miao, Yalin Miao, Chun Xiao, Fang Chen, Dagan Feng

**Affiliations:** 1 Xi’an Jiaotong University City College, 8715 Shangji Road, Xi’an, Shaanxi 710018 People’s Republic of China; 2 DATA61, CSIRO, 13 Garden Street, Eveleigh, 2015 NSW Australia; 3 The University of Sydney, 1 Cleveland Street, Darlington, 2008 NSW Australia; 4 Xi’an University of Technology, 5 Jinhua Nan Road, Xi’an, 710048 Shaanxi People’s Republic of China; 5 Xiangtan University, Xiangtan, 411105 Hunan People’s Republic of China

**Keywords:** Volume rendering, Topology, Automatic illumination design, Distance, Saliency, Perception

## Abstract

**Background:**

Direct volume rendering is one of flexible and effective approaches to inspect large volumetric data such as medical and biological images. In conventional volume rendering, it is often time consuming to set up a meaningful illumination environment. Moreover, conventional illumination approaches usually assign same values of variables of an illumination model to different structures manually and thus neglect the important illumination variations due to structure differences.

**Results:**

We introduce a novel illumination design paradigm for volume rendering on the basis of topology to automate illumination parameter definitions meaningfully. The topological features are extracted from the contour tree of an input volumetric data. The automation of illumination design is achieved based on four aspects of attenuation, distance, saliency, and contrast perception. To better distinguish structures and maximize illuminance perception differences of structures, a two-phase topology-aware illuminance perception contrast model is proposed based on the psychological concept of Just-Noticeable-Difference.

**Conclusions:**

The proposed approach allows meaningful and efficient automatic generations of illumination in volume rendering. Our results showed that our approach is more effective in depth and shape depiction, as well as providing higher perceptual differences between structures.

## Background

Direct volume rendering (DVR) is one of flexible and effective visualization methods for volumetric data. For example, in biological image analysis, 3D microscopic image visualization is widely used to help the user explore biological structures easily and even guide the user to perform smart microscopic imaging interactively [1–3]. Therefore, a volume visualization that can discern and depict more details is highly desirable. In volume rendering, each voxel is usually regarded as a radiance emitter having a certain degree of density, and relies on the optical model of emission and absorption to visualize objects. With the use of transfer functions mapping from voxel values and other data features to different opacities and colors, the structures contained inside the volumetric data can be visualized without the use of any external lighting. After the viewpoint and transfer functions are defined in volume rendering, illumination parameters are the main factors which decide the visual perception of objects in the volume [4]. Effectiveness of visualizing structural details in volume rendering can be affected by both insufficient and excessive illumination [5], and therefore illumination is a significant factor helping improving effectiveness of volume rendering in depicting 3D objects. Local illumination models, such as Blinn-Phong model, mainly help depict local details and the structural shape. On the other hand, global illumination models focus on the depiction of the occlusion relationships between structures with the use of mutual shadowing [4].

Illumination design in volume rendering has been investigated extensively from different perspectives, such as optical model design [6], lighting optimization based on perception [7] and structure [4]. Although different data features (e.g. gradients, scalar values) and illumination settings (e.g. diffuse, ambient, and specular coefficients, position of lighting source) are used, more realistic appearances based on user’s preferences are the main objectives for most of conventional illumination approaches. Since one of the ultimate goals of volume rendering is to get useful insights of volume data, conventional illumination approaches may mislead users focusing on creating colorful images. Moreover, conventional illumination approaches often assign same illumination parameters for all structures, thus miss variations of illumination of different structures. The variations of illumination provide important visual cues for spatial depth under natural lighting [8]. Despite the concept of lighting transfer function [9] trying to vary lighting coefficients of a local illumination model based on gradient information, similar to conventional opacity transfer functions based on gradients [10], it still cannot depict differences of various structures effectively from the illumination perspective. Therefore, in order to depict perception and importance differences between structures from the illumination perspective in volume rendering, a new illumination approach is highly desirable. Moreover, conventional volume rendering often defines illumination variables manually. Such tasks require users have the complex knowledge in computer graphics such as light behaviors and shading models and even require the knowledge in arts [11]. It is often time consuming to define meaningful illumination parameters for visualization even for experienced users.

On the other hand, topology has been widely used in visualization. For example, the contour tree is one of data structures depicting topological relationships of connected isosurfaces/contours [12]. Topology has been used in volume rendering in following aspects: provide topological features of a volume [12]; generate transfer functions in volume rendering [13–15]; and index volumetric data segmentations [16]. Moreover, various topological measures of importance can be used to represent importance of structures [17]. However, no work has been found on how topology is used to control illuminations in volume rendering. All these motivate us to utilize topology in illumination design in volume rendering in order to describe differences between structures in importance and perception with the use of illuminations. The generated illumination parameters are expected to be used to get wide dissimilar rendering outputs and to depict importance relationship between structures. Therefore, measures that are used to control illumination differences between structures based on the topology also need to be defined.

This paper introduces an approach of automating illumination design in volume rendering by utilizing topological features defined by the contour tree of a volume. The objective of this work is to define a new illumination design diagram based on the topology. The generated illuminations can represent differences of structural importance, depict perception differences between neighboring structures, and maximize these differences between structures from the illumination perspective. The proposed approach allows more meaningful and efficient automation of illumination generations, and the exploration on the data can be got from the illumination perspective by the controlling of illumination weighting factors meaningfully instead of complicated adjustments of other physical concepts on illumination. This paper focuses on using topological attributes to define shading parameters such as diffuse coefficients and lighting parameters such as attenuation parameters. We refer to all of these as illumination design.

The contributions of the paper are as follows:

- Topology is introduced into the illumination design and a comprehensive framework is delivered dedicated to generate illumination parameters controlled by topological attributes.
- Based on the framework, topology-aware illumination attenuation is presented which allows dimming out less important structures while keeping more important structures.
- Based on the framework, topology-aware illumination transfer function based on topological saliency and topological distance is presented to depict differences of structural importance from the illumination perspective.
- A two-phase topology-aware illumination perception contrast model is proposed to maximize illumination perception differences between neighboring structures based on the psychological concept of Just-Noticeable-Difference (JND).

## Methods

### Preliminaries

#### Definition of contour trees

The topology of data sets provides a compact and abstract global view that leads to easier and enhanced analysis across applications [18]. The commonly used data structures for explicitly storing topological attributes include: Reeb graphs [19] and Morse-Smale (MS) complexes [20]. The MS complex decomposes the domain of a function into regions which have uniform gradient flow [21]. The Reeb graph [19] is a structure which summarizes the topology of a Morse function. Components of isosurfaces are traced by the Reeb graph as isosurfaces sweep the domain. The Reeb graph is simply connected for functions with simply connected regions, and this graph is also called the Contour Tree (CT). The contour tree is used in this paper for topological representation of data sets.

The isosurface/contour concept is used to define the contour tree. Considering a continuous scalar field F defined on a domain R^d^, *f* : R^d^ → R, R^d^ is defined to be a simplicial complex. The function value of a point inside a simplex is a linear interpolation of values between vertices. The interval between the minimum and maximum values of the function *f*, [*f*_*min*_*, f*_*max*_], is defined as the *functional range* of the field F. Given a value *h* ϵ [*f*_*min*_*, f*_*max*_], the *level set* of the field F at the value *h* is defined as *L(h) =* {*x | f (x) = h*}. The topology of the level set changes only at critical points of F when *h* scans monotonically through the entire range [*f*_*min*_*, f*_*max*_] of F. Contours/isosurfaces appear at local minima of *f*, join or split at saddles, and disappear at local maxima of *f* with the increase of *h* in the level set of *L(h) =* {*x | f (x) = h*}. When each contour is denoted as a node, the level set evolution in the field F forms a tree structure which is called contour tree. It shows the nesting relationships of connected contours/isosurfaces.

The contour tree is typically denoted with a node list and an arc list. Each node of the contour tree corresponds to a critical point of a scalar field. A node pair is defined as an arc. Pascucci et al. [22] proposed branch decomposition approach, and a branch is defined as a monotone path in the tree graph traversing a sequence of nodes.

The noise in the data can add small scale topological features in the contour tree and cause the size of contour tree to increase dramatically [23]. It makes difficult to recognize branches belonging to objects of interest, and results in the contour tree being impractical in data analysis and visualization. Therefore, the contour tree simplification is often conducted to remove unimportant branches and make the size of the tree small enough for the user interaction while maintaining essential structure of the data. Carr et al. [23] simplified the contour tree by defining importance measures with local geometric properties. The typical importance measures include persistence, volume, and hypovolume [23]. Persistence is defined as the absolute difference in scalar values between two critical nodes. Volume is defined as the voxel count of the region enclosed by the isosurface/contour. Hypervolume is the integral of the scalar field over the enclosed region by the isosurface/contour. Zhou et al. [17] integrated multiple measures of importance together to simplify the contour tree. An appropriate simplification of the contour tree helps to improve its capability of indexing objects in data sets and thus improve the rendering quality.

#### Contour tree based data segmentation

**Fig. 1:**
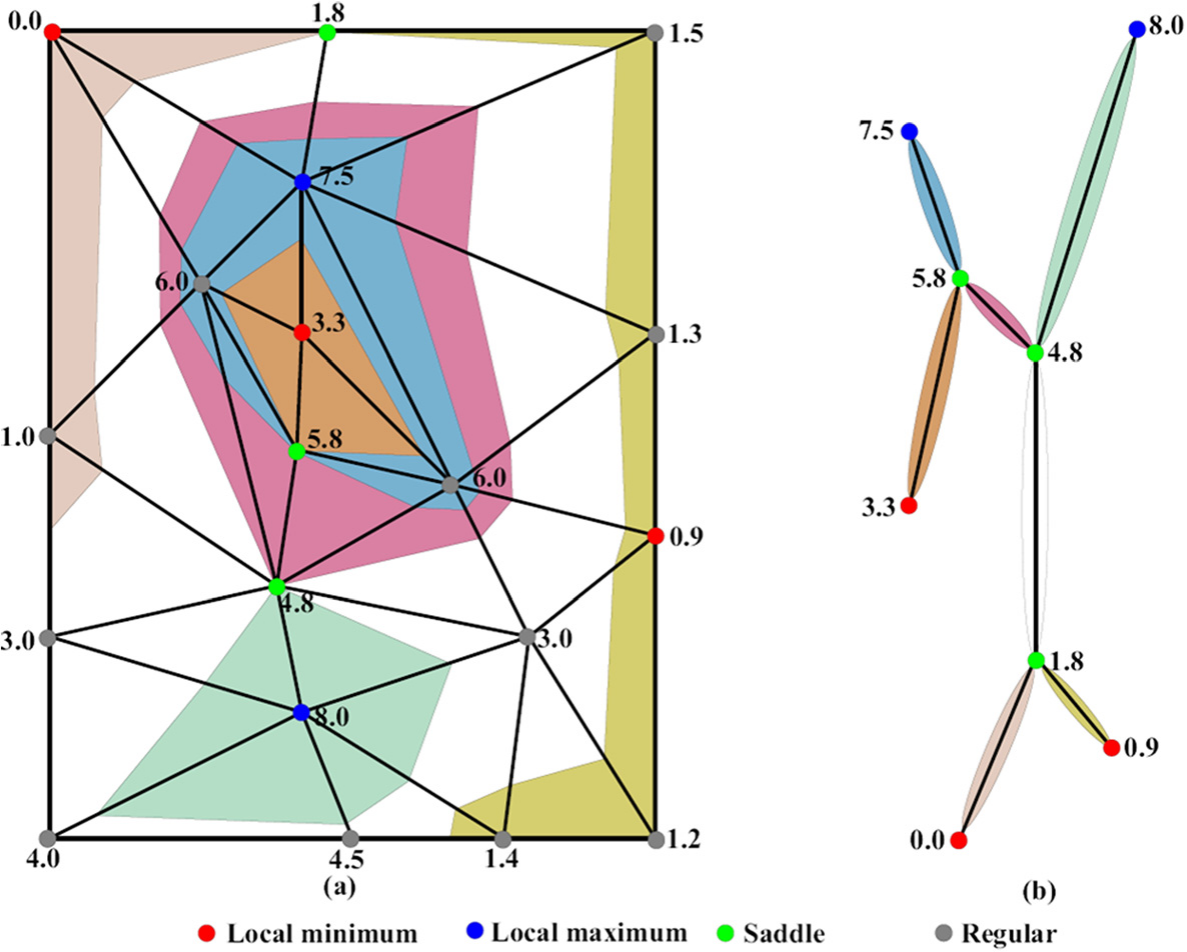
A 2D mesh example and its corresponding contour tree. The 2D mesh is segmented into subregions based on the contour tree

Since the contour tree is set up based on the concept of flexible isosurfaces, the data can be segmented into different regions when isosurfaces sweep through the data [23]. Therefore the contour tree can be considered as a visual index of different zones/subregions in the data and topological relations (e.g. neighboring, inclusion) between subregions are encoded in the contour tree. Figure 1 shows a 2D mesh example and its corresponding contour tree. It presents the segmented 2D mesh with corresponding branches of the contour tree. Branches of the contour tree and their corresponding subregions are encoded with colors. Based on this concept, Weber et al. [14] proposed a volume rendering framework where the contour tree is used to classify volume data and unique transfer function is assigned to each subregion indexed by a branch of the contour tree. Zhou and Takatsuka [15] proposed a model to automatically generate transfer functions for each subregion utilizing the contour tree. The contour tree indexed data segmentation approach makes volume rendering more meaningful by encoding topology of structures in rendering results.

In this paper, besides indexing various subregions, the contour tree is also used to automatically generate different illuminations for subregions with the use of topological attributes derived from the contour tree. The topology-aware illumination design allows users perceive structural differences in various aspects (e.g. importance, topological distance) from the illumination perspective.

#### Illumination models

Different illumination models have been developed for volume rendering. With no loss of generality, we use the classical Blinn-Phong model as the basis of our approach. With the use of the Blinn-Phong model, the color of a voxel in rendered image can be computed as:

1 $$ C=\left({k}_a+{K}_d\left(N\cdot L\right)\right){C}_{tf}+{k}_s{\left(N\cdot H\right)}^n $$

where *k*_*d*_, *k*_*a*_, and *k*_*s*_ are the diffuse, ambient, and specular lighting coefficients respectively, *n* is the shininess exponent, *C*_*tf*_ denotes the color defined by the transfer function, *L* denotes the normalized lighting direction, *N* denotes the normalized gradient vector of the voxel, and *H* denotes the normalized half-way direction. *k*_*a*_ is used to determine how much ambient light is actually reflected. *k*_*d*_ is used to control the amount of diffuse light reflected. *k*_*s*_ is used to control the amount of specular light reflected. *k*_*a*_, *k*_*d*_, and *k*_*s*_ are usually restricted in [0,1].

Ambient reflection is usually used to simulate the ‘radiant’ effect in illumination. It comes from various light sources and is also scattered in various directions. Specular reflection is exhibited from shiny surfaces–it is the reflection of the light source towards the viewer. It is often used to simulate mirror-like reflection. The specular color in this study is defined to be white to preserve the original color from transfer functions. Diffuse reflection is defined to simulate re-emission effects. Of all reflections, diffuse reflection is the most instinctive meaning of the lighting for an object. Therefore, in this paper we focus more on diffuse reflection modulation based on topological features in order to emphasize structures from the illumination perspective.

Real light has an attenuation factor—light intensity becomes weaker over distance. This is similar to the acoustic attenuation, where the perception of sound volume decreases the further away the listener is from its source. In computer graphics, an attenuation factor *f*_*att*_ [24] is often introduced in order to include attenuation into the illumination equation:

2 $$ C=\left({K}_a+{k}_d{f}_{att}\left(N\cdot L\right)\right){C}_{tf}+{k}_s{f}_{att}{\left(N\cdot H\right)}^n $$

Light falloff obeys what is commonly known as the inverse square law, where light’s intensity decreases exponentially in relation to distance.

This paper proposes topology-aware lighting attenuation, where the attenuation factor *f*_*att*_ is defined based on various topological features.

### Topology-aware illumination design

#### Framework overview

In this paper, the effectiveness of topology for the illumination design is investigated from following aspects:

- The topology-aware illumination attenuation is presented to selectively dim out structures based on topological features;
- The topological saliency is used in the lighting transfer function to depict relative structural importance from illumination perspective;
- The topological distance (e.g. topological depth which is defined as the number of the current branch level from the root branch) is incorporated into the lighting transfer function to depict structural differences from distance (depth) perspective;
- The illumination perception contrast is controlled through Just-Noticeable-Difference (JND) based on topological features in order to maximize illumination differences among structures from the perception perspective.

**Fig. 2:**
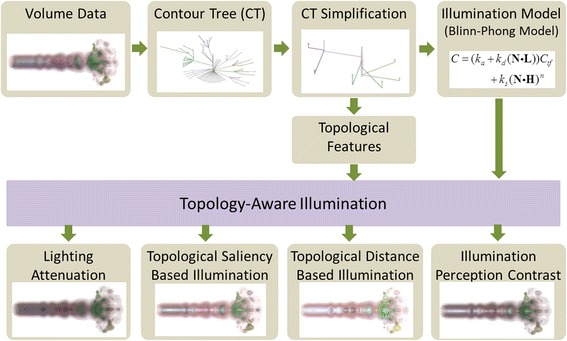
Framework of topology-aware illumination design

The framework of topology-aware illumination design proposed in this paper is illustrated in Fig. 2. In this framework, the contour tree from the original data is firstly simplified to decrease the adverse effect from data noise for topology. The topological attributes based on the contour tree are then combined with the illumination model to define various topology-aware illumination effects. The details of each topology-aware illumination approach are presented in the following subsections.

#### Topology-aware lighting attenuation

Lighting attenuation shows that lighting becomes weaker over distance. The distance is considered in the illumination model with the use of an attenuation factor *f*_*att*_ as shown in Equation 2. In this paper, we adapt the Stokes’ law of sound attenuation to model the lighting attenuation based on topological attributes. The Stokes’ law [25] is the first successful theory of sound attenuation due to the effect of molecular viscosity of the fluid. It models the attenuation of sound in a Newtonian fluid, such as water, due to the fluid’s viscosity [25]. It shows that the amplitude of a plane wave decreases exponentially with traveled distance, at a rate *α* defined by Equation 3:

3 $$ \alpha =\frac{2\eta {\xi}^2}{3\rho {V}^3} $$

where *α* is also called the attenuation coefficient, *η* denotes the dynamic viscosity coefficient of the fluid, *ξ* denotes the frequency of the sound, *ρ* denotes the density of the fluid, and *V* denotes the speed of sound in the fluid. The attenuation coefficient *α* characterizes how easily a beam of light, sound, particles, or other energy or matter can penetrate a material or medium. A large value of attenuation coefficient corresponds to the situation that the beam is quickly ‘attenuated’ (weakened) when it transmits through the medium.

Similarly, as the light transmits through a medium such as the water, the shortwave radiation is attenuated by the medium, and the intensity of light decreases exponentially with the distance passed by the light. The intensity of light at distance *z* can be calculated using the Beer-Lambert Law [26]:

4 $$ {I}_z={I}_0{e}^{-\alpha z} $$

where *I*_*0*_ is the intensity of the incident light, *Iz* is the light intensity at distance *z* in the medium, *α* is the attenuation coefficient. Based on these preliminaries, the lighting attenuation factor *f*_*att*_ in this study is defined as in Equation 5:

5 $$ {f}_{att}={e}^{-\alpha z} $$

In this study, the light is modeled to pass through an isotropic and homogeneous medium such as water during rendering. Stokes’ law is applied to the light propagation to get attenuation coefficient *α* based on topological attributes. Based on this idea, the distance *z* in Equation 5 is modelled as the persistence *p* of branches. The light is assumed to pass through the water. Therefore, various parameters in Equation 3 is modelled as follows: water at 20 °*C* has a viscosity *η* of 0.001002 *P∙as*, the density *ρ* at 20 °*C* is 998.2071 *kg/m*^*3*^. The frequency *ξ* is modelled as the number of siblings *n*_*i*_^*s*^, the Speed *V* represents the lighting speed in the medium and controlled by the user interactively. The sibling branch refers to the one which has the same parent branch as the current branch.

In this topology-aware illumination attenuation, *f*_*att*_ has the inverse relation with topological features of persistence *p* and number of siblings *n*_*i*_^*s*^. Therefore, it allows the option of dimming out less important structures while keeping more important structures. As a result, important structures are emphasized from the illumination perspective.

#### Topology-aware lighting transfer function

Lum and Ma [9] introduced lighting transfer function (LTF) to define different lighting coefficients of *k* values in the illumination model as in Equation 1. Following the LTF concept, this section presents a method of topology-aware lighting transfer function, which incorporates topological attributes into the LTF. In this method, *k*_*a*_, *k*_*d*_, and *k*_*s*_ in Equation 1 are defined as functions of topological features for different regions corresponding to branches in topology-aware lighting transfer functions. The overall objective of topology-aware lighting transfer functions is to depict structural differences from illumination perspective in order to identify various objects easily and effectively.

In this subsection, topology-aware lighting transfer function is defined based on two aspects:

- Topological saliency. Lighting coefficients (*k*_*a*_, *k*_*d*_, and/or *k*_*s*_) for each region are defined as functions of topological saliency (relative importance of structures).
- Topological distance. Lighting coefficients for each region are defined as functions of topological distance.

#### Topological saliency-based illumination

We use an approach based on topological saliency [27] to evaluate relative importance of branches. Let the set *B* = {*b*_*1*_, *b*_*2*_, …, *b*_*n*_} be the set of branches in the contour tree, *t*_*i*_ is the topological feature created at *b*_*i*_, *d*(*b*_*i*_,*b*_*j*_) is the distance between two branches of *b*_*i*_ and *b*_*j*_. Consider a *r*-neighborhood *Nr(i)* = {*x ϵ B*| *d(x,b*_*i*_*) ≤ r*}, which is the ball of radius *r* centred at branch *b*_*i*_. The topological saliency depicts relative importance of branches locally.

The topological saliency *S*_*i*_^*r*^ of the feature created at *b*_*i*_ is defined as:

6 $$ {S}_i^r=\frac{\omega_i^i{t}_i}{{\displaystyle \sum_{b_j\in B}{\omega}_j^i{t}_j}} $$

where *t*_*i*_ can be persistence *p*_*i*_ or volume *v*_*i*_ of branches. *ω*_*j*_^*i*^ is a Gaussian weighting function for the feature *j* with respect to *i*:

7 $$ {\omega}_j^i={e}^{-\frac{d{\left({c}_i,{c}_j\right)}^2}{r^2}} $$

In order to make inner structures more clear relative to outmost structures, this paper focuses on the topological saliency of structures between outmost structures and inner structures. Therefore, *d(c*_*i*_,*c*_*j*_*)* in Equation 7 is defined as the distance between the root and children branches, more specifically, the topological depth difference between branches is defined as the distance between two branches. *r* is defined as the maximum topological depth in the contour tree.

From the illumination model as shown in Equation 1, *k*_*a*_, *k*_*d*_, and *k*_*s*_ are used to control the contribution of various reflections in the illumination model. Furthermore, as mentioned, the most instinctive illumination of an object is the diffuse lighting. Therefore, in order to introduce the topological saliency into the illumination design in this section, we define the diffuse reflection coefficient *k*_*d*_ as a function of topological saliency. This approach is also called saliency illumination in this paper.

8 $$ {k}_d=f\left({S}_i^r\right) $$

where *f* is the function of the saliency *S*_*i*_^*r*^ of a branch, for example, *k*_*d*_ can be defined as:

9 $$ {k}_d=w{S}_i^r $$

where *w* is the weight modulated by the user. In this saliency-based illumination model, higher illumination is defined to objects which are relatively more important by assigning higher *k*_*d*_ to them. *k*_*a*_ and *k*_*s*_ are pre-defined and have the same value for all structures. Therefore, the variations of illumination of objects are primarily determined by *k*_*d*_, which reflects topological importance of structures.

#### Topological distance-based illumination

Besides topological saliency, topological distance of objects also plays significant roles in information depiction in volume rendering. This subsection introduces the topological distance, which is defined as the topological depth in this paper, into the topology-aware illumination design in order to highlight inner objects from the topological distance perspective. This approach is also called distance illumination in this paper.

Because of properties of the monotonicity and finite range of (0, 1) of a Sigmond function, it is used to introduce topological distance into the illumination design in this paper. This paper defines *k*_*d*_ as a function of topological distance with Sigmond function in Equation 10.

10 $$ {k}_d=w\frac{1}{1+{e}^{-z}} $$

where *w* is the weight interactively modulated by the user, *z* is the topological distance, such as the depth of a branch. In this illumination model, the objects with higher topological depth are assigned with higher *k*_*d*_, which means that inner structures have high illuminance in order to highlight them.

### Topology-aware illumination perception contrast model

#### Overview

Illumination design has been regarded as one of significant factors in conveying visual cues for 3D object perception. This subsection presents a topology-aware illumination perception contrast model which utilizes topological features to maximize illuminance visual perception differences between neighboring objects (also called as perceptual illumination).

Naturally, when light is coming to a semi-transparent object, the outer surface gets high illuminance and inner surface gets less illuminance, which makes inner objects obscure and difficult for users to understand. Therefore, in order to make inner objects more visible in a data set, less illuminance is applied to outside structures while higher illuminance is applied to inner structures. From illumination model perspective as shown in Equation 1, higher values of *k*_*a*_, *k*_*d*_, and/or *k*_*s*_ need to be defined to inner structures than that to outside structures. The reason that why this approach works can be obtained by imagining that an object inside a semi-transparent box: *it is usually easier for users to perceive and better understand the glittery object inside a semi-transparent box than the dark object inside a glittery box*.

Before the discussion of details of the topology-aware illumination perception contrast model, two concepts are firstly introduced in this subsection: illumination contrast ratio and Just-Noticeable-Difference.

Illumination Contrast Ratio: it is a factor which describes the degree of illumination difference between two regions. It is defined as in Equation 11:

11 $$ \gamma =\frac{L_i}{L_j} $$

where *γ* is the illumination contrast ratio between branch *i* and *j*. In this study, the illumination for the parent branch is usually lower than illumination for child branches in order to emphasis inner structures. *γ* is the illumination ratio between parent branch and child branch. Therefore, the range of *γ* is [0,1], where *γ* = 0 corresponds to the situation where the lower illumination is black, while *γ* = 1 means that the two branches have the same luminance. In our implementation, *γ* is allowed to be interactively changed in order to let users learn how illumination affects the perception of visualization.

Just-Noticeable-Difference (JND): it is a frequently used measure of perceptibility [7, 28], which is the minimal change of luminance required for an observer to gain a perception difference. The relationship between JND Δ*L*_*i*_ and luminance *L*_*i*_ is well studied in psychophysics [29]. Ferwerda et al. [30] and Larson et al. [31] used a function for the whole human vision range to depict the relation between the JND Δ*L*_*i*_ and the luminance *L*_*i*_ as formulated in Equation 12:

12 $$ \begin{array}{l} \log \left(\varDelta {L}_i\right)=\\ {}\left\{\begin{array}{c}\hfill \begin{array}{c}\hfill \begin{array}{c}\hfill -2.86,\kern4em  if\  log\left({L}_i\right)<-3.94\kern4em \hfill \\ {}\hfill {\left(0.405\times \log \left({L}_i\right)+1.6\right)}^{2.18}-2.86,\kern5em \hfill \end{array}\hfill \\ {}\hfill \kern5.75em \mathrm{if}\ \hbox{-} 3.94\le \log \left({L}_i\right)<-1.44\hfill \\ {}\hfill \log \left({L}_i\right)-0.395,\kern0.5em \mathrm{if}\ \hbox{-} 1.44\le \log \left({L}_i\right)<-0.0184\hfill \end{array}\hfill \\ {}\hfill {\left(0.294\times \log \left({L}_i\right)+0.65\right)}^{2.7}-0.72,\kern4.5em \hfill \\ {}\hfill \kern5.75em \mathrm{if}\kern0.5em \hbox{-} 0.0184\le \log \left({L}_i\right)<1.9\hfill \\ {}\hfill \log \left({L}_i\right)-1.255,\kern0.5em \mathrm{if} \log \left({L}_i\right)\ge 1.9\kern5.25em \hfill \end{array}\right.\end{array} $$

Wang et al. [7] enhanced visual perception of 3D volumetric objects using various types of directional lights. JND in illumination is used to measure illumination differences between objects. However, Because of the lacking of topological information in the visual perception enhancement, it does not enhance topological relations between objects from the illumination perspective. Our target is to optimize the Δ*L*_*i*_ based on topological features in order to maximize illumination differences between neighboring structures.

In this subsection, a two-phase approach is proposed for topology-aware illumination perception contrast model:

- Initialization Phase: Initialize illumination for each branch based on the JND which is the minimum luminance difference between branches;
- Optimization Phase: Optimize the luminance difference between branches in order to maximize luminance differences between branches.

#### Initialization phase

This phase initializes luminance of each branch based on the JND. In this phase, the luminance *L*_*0*_ for the root of the contour tree is firstly specified by the user. Then the luminance difference *ΔL*_0_^*JND*^ between the root and its children is calculated based on the JND with Equation 12. The initial luminance of child branches of root is *L*_*c*_ = *L*_*0*_ + *ΔL*_0_^*JND*^. The illumination of other branches is recursively defined in a similar way from root to other branches until illumination is defined for all branches.

After finishing the initialization phase, the illumination is defined for each branch which has the minimum luminance difference with its neighboring branches.

#### Optimization phrase

The objective of optimization phase is to optimize luminance difference between neighboring branches to maximize luminance JND based on topological properties. In this phase, a luminance contrast ratio *γ*_*0*_ between root and its child branches is firstly defined. Based on *γ*_*0*_ and *L*_*0*_ defined at the initialization phase, a combination of JND and contrast ratio is used to optimize luminance differences between branches. This paper focuses on the luminance contrast between parent and child branches because this contrast is one of significant factors that affect the visualibility of inner structures. This process is shown in Table 1. The optimization includes two processes: illumination distribution from parent to children, and illumination distribution among siblings.

**Table 1:**
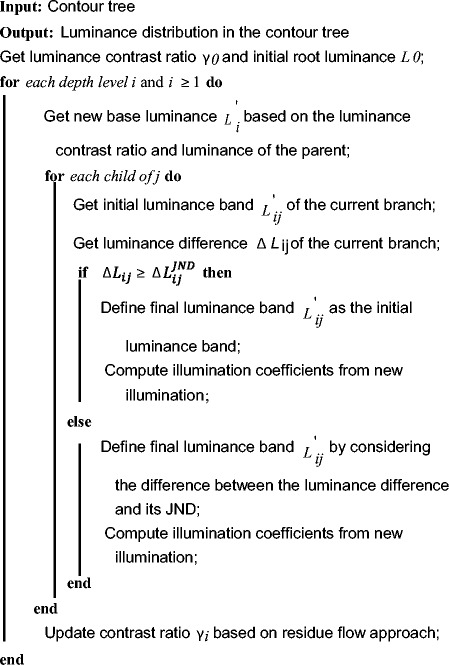
Luminance difference distribution in the contour tree

##### Illumination distribution from parent to children

The optimisation phase begins from the root. The new base luminance of root’s children is got based on the *γ*_*0*_ and *L*_*0*_:

13 $$ {L}_1^{\hbox{'}}={L}_0/{\gamma}_0 $$

where *L*_1_^'^ is the new base luminance of child branches of the root. In general, given the luminance contrast ratio *γ*_*i−1*_, the new base luminance on the *i*th level is:

14 $$ {L}_i^{\hbox{'}}={L}_{i-1}/{\gamma}_{i-1} $$

The optimization needs to meet conditions on each depth level as formulated in Equation

15 $$ \Big\{\kern1em \begin{array}{c}\varDelta {L}_i\ge \varDelta {L}_i^{JND}\kern1em \\ {}{\gamma}_i\ge {\gamma}_0\kern1em \\ {}{L}_i^{{\textstyle \hbox{'}}}\ge {L}_i\end{array}\kern1em \operatorname{} $$

where *ΔL*_*i*_ is the luminance difference between branches, *ΔL*_*i*_^*JND*^ is the luminance JND between branches based on Equation 12, *L*_*i*_ is the luminance applied in the initialization phase based on JND. All these conditions guarantee that each object has optimized luminance JND and high luminance contrast ratio with its neighboring structures in order to differentiate objects from the illumination perspective effectively.

##### Illumination distribution among siblings

The base illumination from the parent branch on each depth level is distributed further between siblings on that level. This further illumination distribution maximizes differences between sibling branches. Various attributes of branches are used to maximize the sibling branch differences: 1) The saddle value of each sibling branch as it determines its exact location on the depth level it resides; 2) Topological importance values of volume, persistence, and hypervolume of each branch. Similar with opacity transfer function generations in [15], the luminance applied to each branch is got with Equation 16:

16 $$ {L}_{ij}^{\hbox{'}}={L}_i^{\hbox{'}}\cdot {f}_b\left({p}_{ij},{v}_{ij},h{v}_{ij}\right)\cdot {f}_d\left({s}_{ij}\right),\kern1em \left(j=1,\dots, {n}_i^s\right) $$

where *L*_*ij*_^'^ represents the new luminance applied to the *j*th sibling on the *i*th depth level. *L*_*i*_^'^ represents the base luminance on the *i*th depth level computed with Equation 14. *v*_*i j*_, *p*_*i j*_, and *hv*_*i j*_ are volume, persistence, and hypervolume respectively of the *j*th sibling branch on the *i*th depth level. *f*_*b*_ denotes the function used to control illumination distribution among siblings based on importance values *p*_*i j*_, *v*_*i j*_, and *hv*_*i j*_. *n*_*i*_^*s*^ represents the number of siblings on the *i*th depth level. *f*_*d*_ represents the function to control the illumination distribution among siblings according to saddle value *s*_*i j*_.

In this paper, *f*_*b*_ is defined as the area of the importance triangle decided by persistence, volume, and hypervolume as presented [17], in order to consider various types of importance at the same time in the illumination computation.

In order to define the function *f*_*d*_ in Equation 16, saddle values of sibling branches are considered. Since each branch is defined as a concatenation of a list of arcs in the general contour tree, the saddle value of a branch reflects the distance between the current branch and the root, which implies inner/outer structural relationships. In accordance with this observation, in order to emphasize inclusion relationship and also emphasize branches with larger saddle values, smaller luminance is applied to lower saddle value branches and larger luminance is applied to larger saddle value branches. Therefore, the function *f*_*d*_ in Equation 16 is defined by Equation 17:

17 $$ {f}_d\left({s}_{ij}\right)=\frac{s_{ij}-{s}_i^{\min }}{\varDelta {s}_i} $$

where $$ \begin{array}{c}\hfill \varDelta {s}_i={s}_i^{\max }-{s}_i^{\min },\hfill \\ {}\hfill {s}_i^{\max }= max\left({s}_{i0},{s}_{i1},\dots, {s}_{i,{n}_i^s}\right),\hfill \\ {}\hfill {s}_i^{\min }= min\left({s}_{i0},{s}_{i1},\dots, {s}_{i,{n}_i^s}\right).\hfill \end{array} $$

After getting *L*_*ij*_^'^, Δ*L*_*ij*_ is calculated with Equation 18. Then the condition of *ΔL*_*ij*_ ≥ *ΔL*_*i*_^*JND*^ needs to be checked before updating the illumination parameters, where *ΔL*_*i*_^*JND*^ is the luminance JND between current branch and its parent. If the condition is not met, the final *L*_*ij*_^'^ is got with Equation 19.

18 $$ \varDelta {L}_{ij}={L}_{ij}^{\hbox{'}}-{L}_{i-1} $$

19 $$ {L}_{ij}^{\hbox{'}}={L}_{ij}+\left|\varDelta {L}_{ij}-\varDelta {L}_{ij}^{JND}\right| $$

##### Luminance contrast ratio update

The luminance contrast ratio *γ*_*i*_ between structures is defined to emphasize inclusion relationships. Structures on higher depth levels (inner structures) are defined with higher contrast (lower *γ*_*i*_ values) in order to emphasize inner structures. The residue flow model proposed for the opacity transfer function definition [15] is adapted to control the updating of luminance contrast ratio *γ*_*i*_ on different depth levels during the luminance distribution. According to the residue flow model in [15], the absolute head loss value Δ*hi* of contrast ratio between the two ends of the current branch is modelled with Equation 20:

20 $$ \varDelta {h}_i=\frac{1}{K}\cdot \frac{Q\cdot {p}_i}{n_i^c} $$

where Δ*h*_*i*_ ϵ [0.0,1.0], *p*_*i*_ denotes the persistence of the branch *i*, *n*_*i*_^*c*^ denotes the number of children of the branch *i. Q* is the fluid flow speed. *K* is a constant and it is defined as the hydraulic conductivity, which is a function of both the particular fluid and the permeability of the porous medium. The sand is used as the porous medium in this study, and *K* is defined as 300 for the sand when water flows through it [32].

In this paper, Δ*h*_*i*_ is used as a factor to modulate luminance contrast ratio *γ*_*i*_ absorbed by the current branch and the residue flowing to the next level of depth of the contour tree. The residue of *γ*_*i*_ is computed by Equation 21:

21 $$ {\gamma}_i=\left(1.0-\varDelta {h}_i\right)\cdot {\gamma}_{i-1} $$

where *i* ≥ 1, Δ*h*_*i*_ ⋅ *γ*_*i−1*_ is the contrast ratio residue created on the *i*th depth level. As described in the previous sections, branches on the lower depth level in the contour tree correspond to outer structures, and branches on the higher depth level in the contour tree correspond to inner structures. Therefore, the residue flow of contrast ratio in the contour tree results in the definition of smaller luminance contrast ratio (higher luminance to child branches) to inner structures, which emphasizes inner structures in volume rendering.

### Evaluation method

#### Hypotheses

Our method was evaluated from three aspects: 1) depth information; 2) shape information; 3) structure difference perception. Therefore, the following hypotheses were posed:

- Topology-aware illumination would help users more easily percept depth information of structures (H1);
- Topology-aware illumination would help users better understand shape information of structures (H2);
- Topology-aware illumination would show more structural difference perception (H3).

#### Evaluation setup

We designed a user study to test hypotheses. Six illumination methods of conventional illumination (CVL), constant diffuse (CSL), importance illumination (IML), distance illumination (DIL), saliency illumination (SAL), perceptual illumination (PEL) were used to render volumetric data. Participants were required to compare effectiveness of various methods in three aspects: 1) depth information; 2) shape information; 3) structural difference perception. A total of 8 participants were recruited in the pilot study. Ages of participants range from early twenties to forties. Participants were postgraduates and researchers from visualization, medical image analysis as well as other computing related fields. The participants were told that there were no risk and discomfort in completing the survey. They were also told that the results of the study may be published at a conference or in a journal article. The participants were told that they may stop the survey at any time when they do not want to continue with any reason. Any information or personal details gathered in the course of the study were confidential and completely anonymized.

Participants were firstly required to view rendering images from different illumination methods. Then they were asked to answer questionnaires to rate each illumination method using a 9-point Likert scale (9 = totally agree, and 1 = totally disagree) on three aspects of each rendering image: depth perception; shape perception; structure difference perception.

## Results

### Experimental results

Experiments on various data sets were conducted in this paper to demonstrate the effectiveness of the presented approach in volume rendering. The experiment was conducted on Ubuntu 14.04 on a MacbookPro machine (Intel Core i5 2.53 GHz, 2G RAM, NVIDIA GeForce GT 330 M graphics card).

**Fig. 3:**
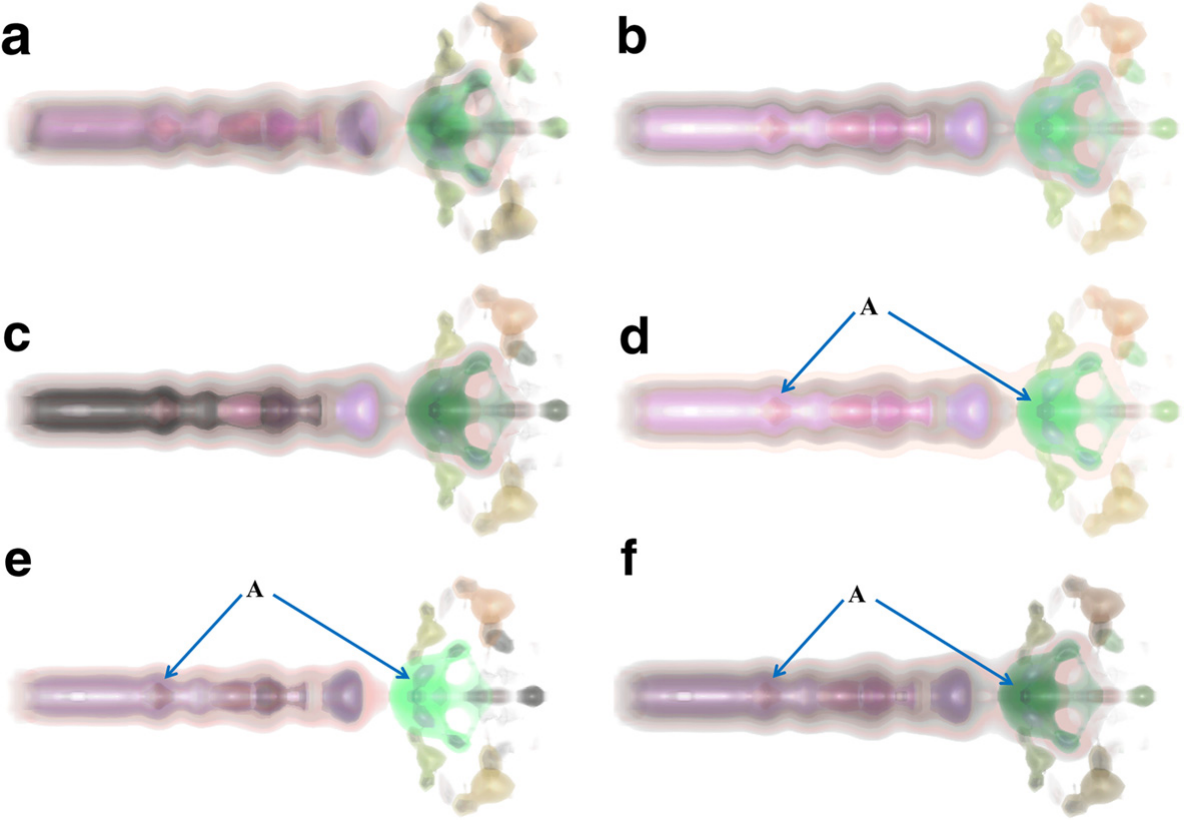
Volume rendering of fuel data set with various illumination approaches: **a** Conventional illumination, **b** Constant illumination, **c** Importance illumination (persistence, *w* = 0.71), **d** Distance illumination (persistence, *w* = 0.71), **e** Saliency illumination (persistence, *w* = 0.71), **f** Perceptual illumination

In this paper, the initial transfer function of data sets is defined based on the topology-controlled transfer function approach presented in [15]. The proposed approach was firstly used to render the ‘fuel’ data set (see http://www9.informatik.uni-erlangen.de/External/vollib/), a volumetric data from a simulation of fuel injected into a combustion chamber. In this paper, the proposed approaches (distance illumination, saliency illumination, and perceptual illumination) were compared with other approaches, e.g. illumination only with ambient and diffuse illumination (called conventional illumination); illumination with same settings of ambient, diffuse and specular lighting for all structures (called constant illumination), and topological importance based illumination which defines ambient coefficient as persistence or volume and keeps other lighting coefficients as constant [33]. From the comparison, we see that all renderings (Fig. 3d–f) from approaches presented in this paper show internal structures more clearly (e.g. structures pointed by A) than other approaches by emphasising internal structures from the illumination perspective. Furthermore, renderings from saliency illumination and perceptual illumination show more obvious differences between structures from illumination perspective. Structures in Fig. 3f show larger illumination differences among structures than renderings in other figures. This is because that perceptual illumination considers the luminance JND and optimises the illumination differences between structures.

**Fig. 4:**
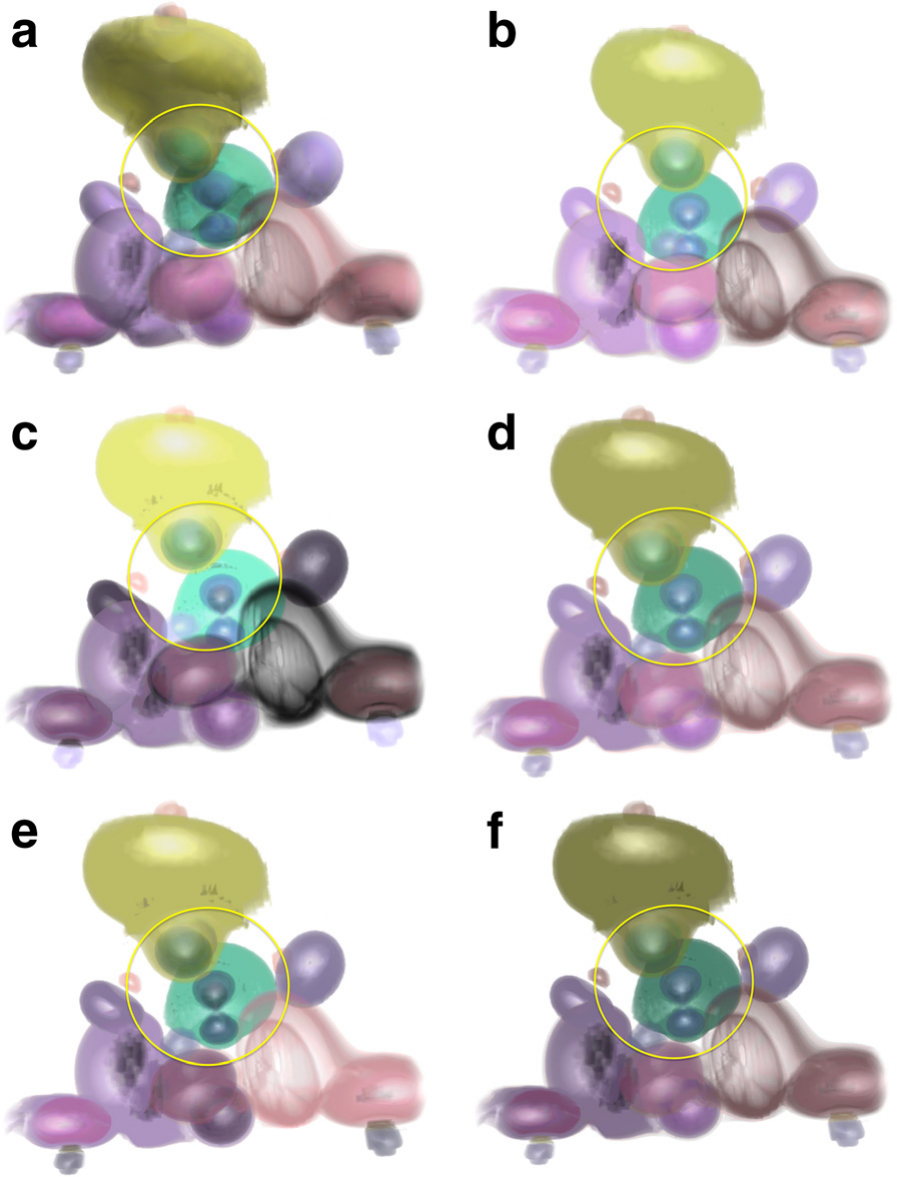
Volume rendering of neghip data set with various illumination approaches: **a** Conventional illumination, **b** Constant illumination, **c** Importance illumination (persistence, *w* = 0.78), **d** Distance illumination (persistence, *w* = 0.57), **e** Saliency illumination (persistence, *w* = 0.67), **f** Perceptual illumination

**Fig. 5:**
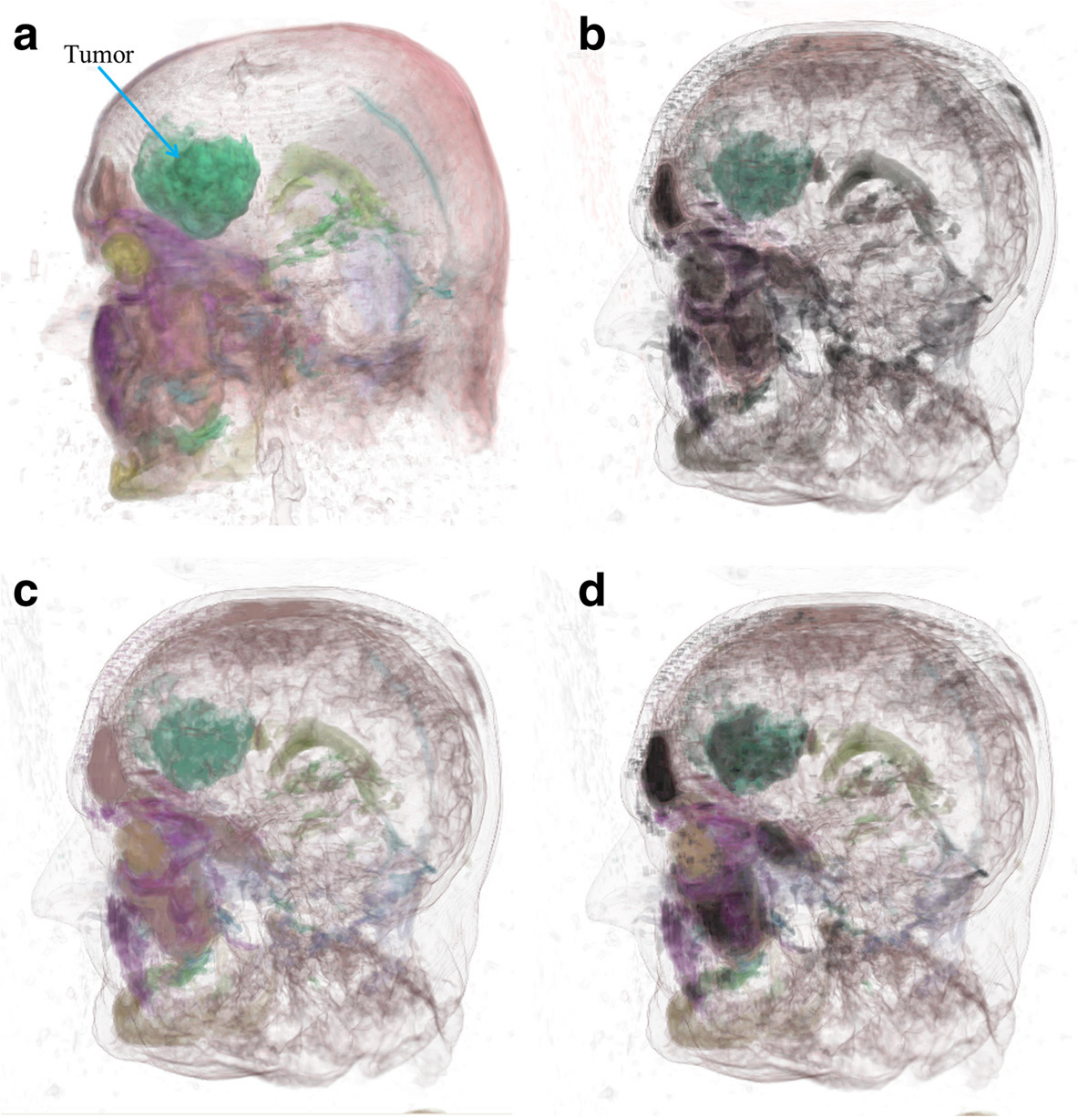
Volume rendering of tumor head data set with various illumination approaches: **a** Conventional illumination, **b** Importance illumination (volume, *w* = 0.28), **c** Distance illumination (volume, *w* = 0.5), **d** Perceptual illumination

Our approach was also applied to render the ‘neghip’ data set (see http://www9.informatik.uni-erlangen.de/External/vollib/), a volumetric data from a simulation of the spatial probability distribution of the electrons in a high potential protein molecule. Figure 4 shows the comparison of renderings using various approaches. Similar conclusions as in Fig. 3 were got in this comparison. For example, for structures in yellow circles, renderings from topological illumination proposed in this paper show more clear internal structural information than other approaches. More obviously, objects rendered in Fig. 4f show higher perceptual contrast in illuminations than other figures, which allows users more easily detect structural differences and structural details. The proposed approach was also applied to a more complicated data set, an MR head data set with brain tumors inside (data courtesy of B Terwey, Bremen). In the MR head data set, brain tumors are often difficult to visualize because of inclusions of tumors inside the brain and complicated brain structures. From the comparison in Fig. 5, we see that topological illumination shows more structural details such as skin surface and brain shapes (Fig. 5b to Fig. 5d) despite conventional illumination also depicting tumours clearly. It is found that perceptual illumination as shown in Fig.5d creates higher and balanced perceptual contrast between objects than other illumination methods.

**Fig. 6:**
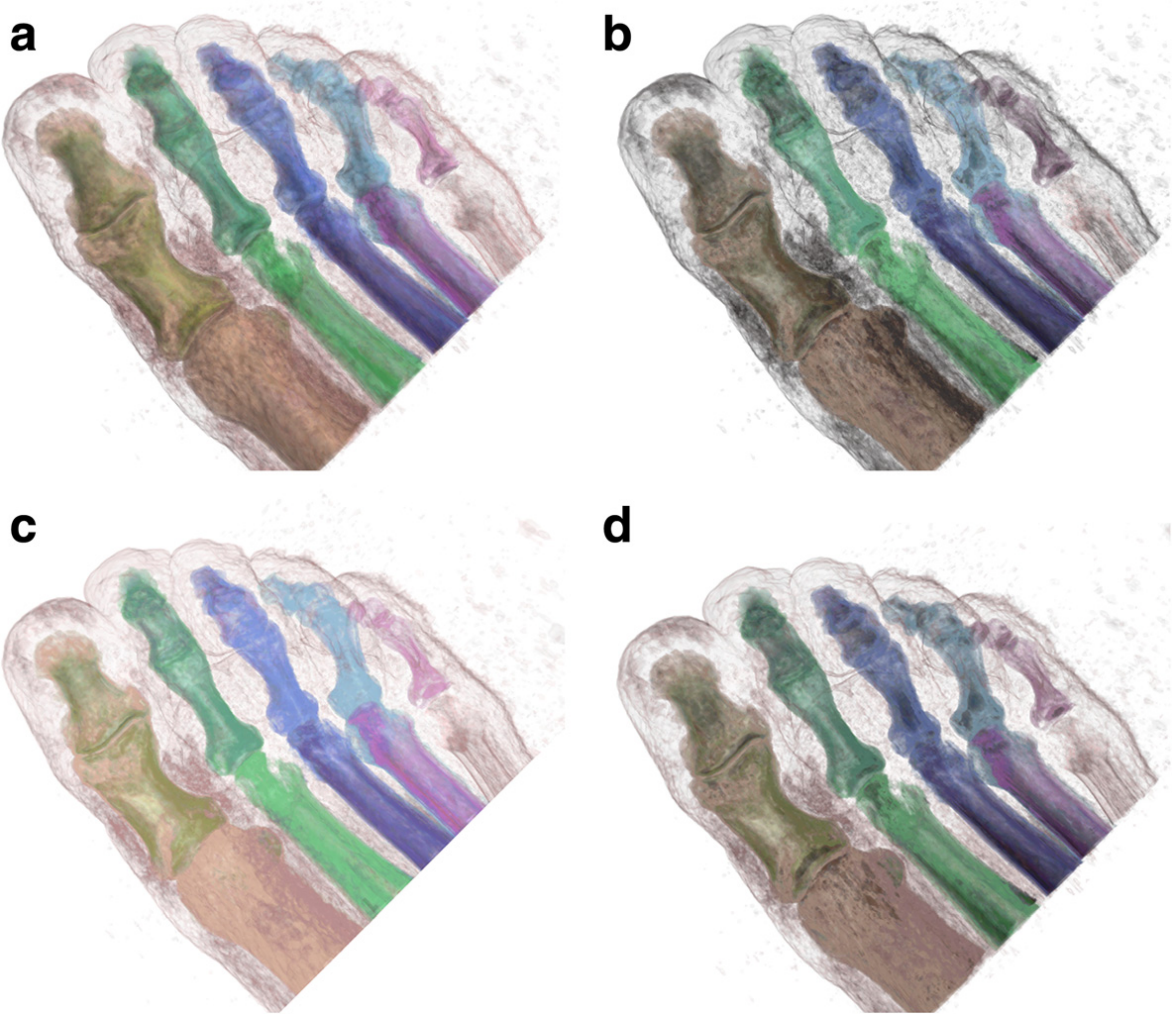
Volume rendering of foot data set with various illumination approaches: **a** Conventional illumination, **b** Importance illumination (volume, *w* = 0.65), **c** Distance illumination (volume, *w* = 0.65), **d** Perceptual illumination

**Fig. 7:**
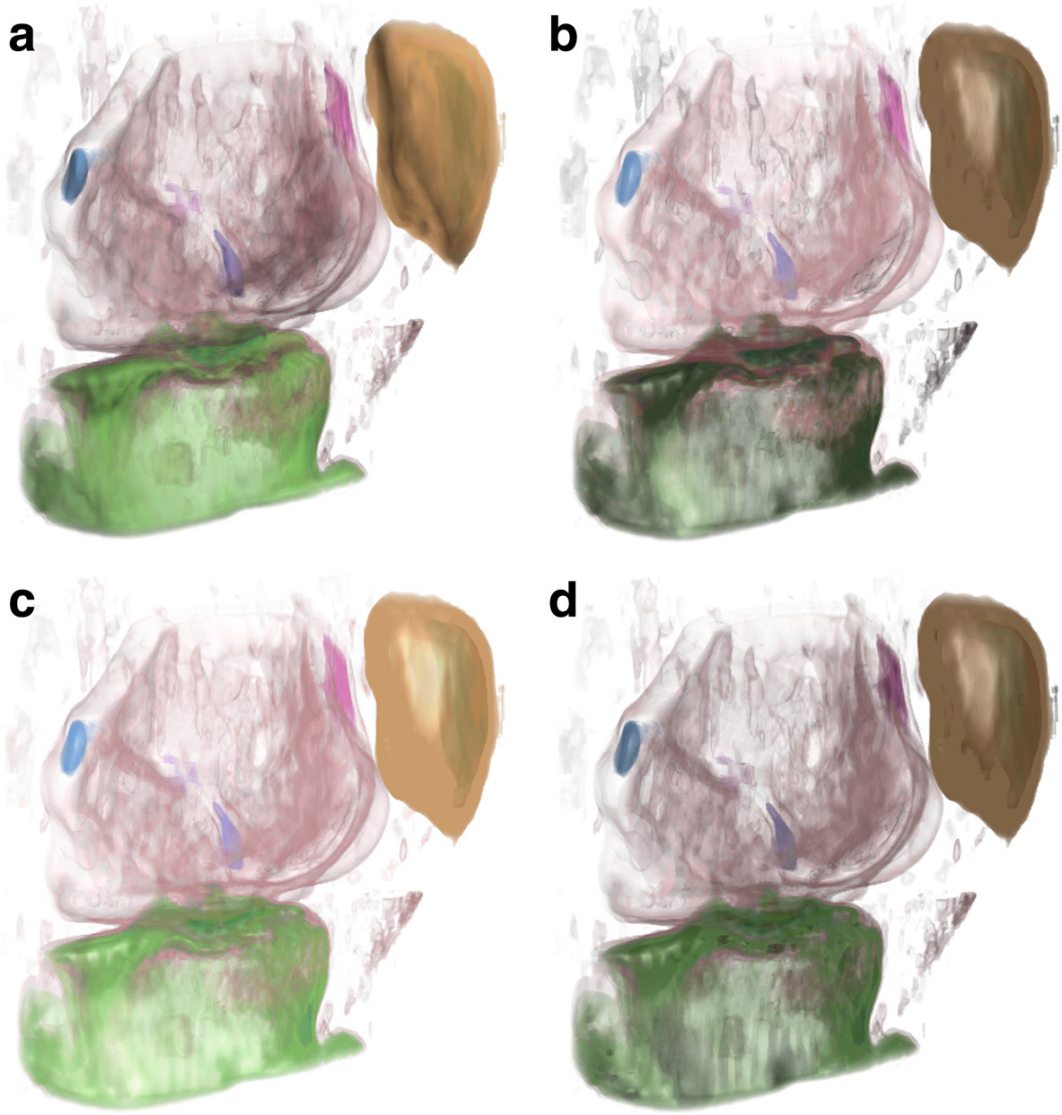
Volume rendering of CT-Knee data set with various illumination approaches: **a** Conventional illumination, **b** Importance illumination (persistence, *w* = 0.76), **c** Distance illumination (persistence, *w* = 0.76), **d** Perceptual illumination

The proposed approach was applied to other medical data sets, CT foot data set and CT knee data set (both are from http://www9.informatik.uni-erlangen.de/External/vollib/) as illustrated in Figs. 6 and 7 respectively. From these results, we see that topological illumination allows emphasise structures based on topological features. For example, compared with conventional illumination, importance illumination emphasises structures which have high volume (thumb bones in Fig. 6b), and high persistence (kneecap in Fig. 7b). Perceptual illumination enhances the perception differences between structures (see Figs. 6d and 7d).

**Fig. 8:**
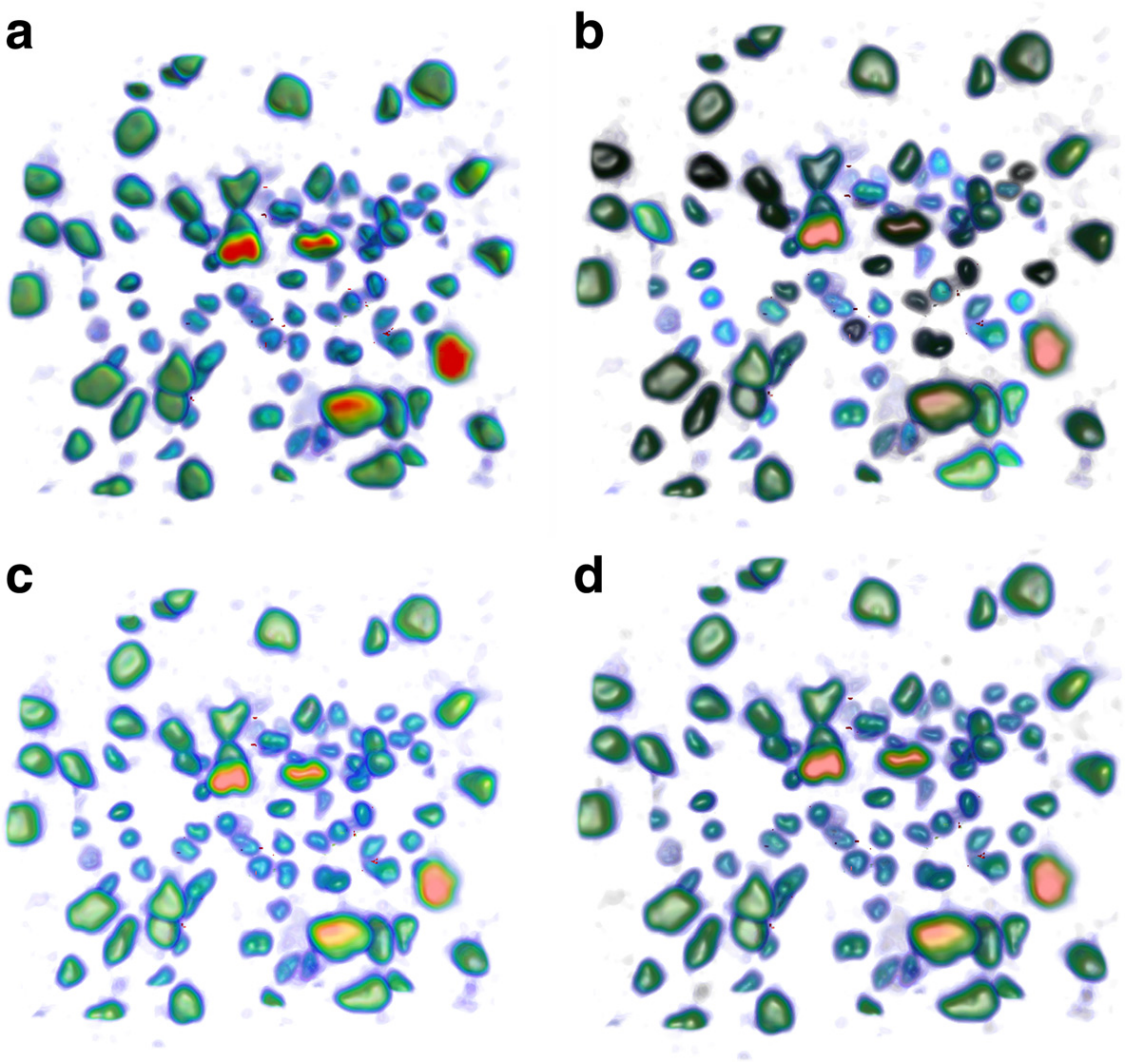
Volume rendering of Cell data set with various illumination approaches: **a** Conventional illumination, **b** Importance illumination (volume, *w* = 0.74), **c** Distance illumination (volume, *w* = 0.74), **d** Perceptual illumination

We also applied our approach to a biological image data set. Figure 8 shows the rendering results of biological cell data set (test data from Vaa3D [1]) with different illumination approaches. Similar conclusions as medical image renderings are got for biological image renderings. For example, compared with conventional illumination in Fig. 8a, importance illumination in Fig. 8b emphasises cells based on the cell volume (voxel count of a cell), which allows user to easily detect volume differences of cells via illumination variations. Figure 8c emphasises cells based on the topological depth, which allows users to percept topological differences of cell structures. Figure 8d enhances the perception differences between cells so that users easily identify different cells. These results show that the proposed approach helps users perceive topological differences of biological data from illumination perspective and thus improve the overall understanding of the biological data set.

**Fig. 9:**
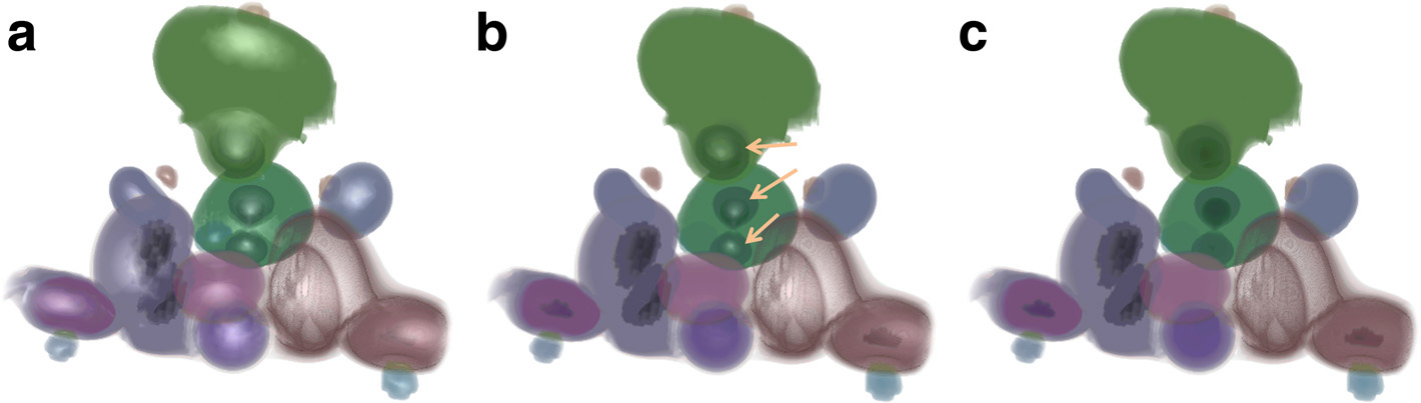
Topology-aware lighting attenuation of neghip data set: **a** *V* = 229, **b** *V* = 154, **c** *V* = 42

We tested the effectiveness of topological attenuation in structural depiction in volume rendering. Figure 9 shows the topological attenuation of neghip data set. The data is originally rendered with constant illumination approach. When topological attenuation is applied, we can see from the figure that object lighting is dimmed out with the decrease of lighting speed *V* from Fig. 9a–c. However, different from the conventional lighting attenuation approach which applies same attenuation factor to objects based on distance only, topological attenuation also considers topological features which can be seen from Fig. 9b. In Fig. 9b, objects pointed by arrows are not dimmed out as other structures because of their high persistence.

The volume rendering pipeline is implemented with the use of GPU (Graphics Processing Unit) fragment programs in this paper. Our system performance allows the real-time exploration of volumetric data. The size of the data set and the branch number of the contour tree determine the processing time of automatically generating illumination parameters. For a small data set, the number of branch of the contour tree is usually within a manageable size (e.g. less than 30 after the contour tree simplification for the ‘fuel’ data set). We can interactively generate illumination parameters for such kind of data sets. We got the frame rate of 36.5fps for the “fuel” data set (20 branches after simplification, and the original branch size is 86).

### Evaluation results

**Fig. 10:**
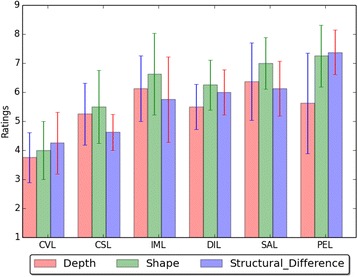
Average subjective ratings of depth, shape and structural difference for different illumination methods in the rendering

We performed Friedman test and post-hoc analysis using Wilcoxon signed-rank tests to analyze the mean differences in participant responses. Post-hoc analysis was performed with a Bonferroni adjusted alpha level set at *α* = .025(.05/2 = .025). This adjusted significance alpha level of .025 was calculated by dividing the original alpha level of .05 by 2 based on the fact that for all tasks we have two conditions to test in general (with/without topological information for illumination computation). Figure 10 shows average subjective ratings of depth, shape and structural difference for different illumination methods in the rendering.

For depth perception, a statistically significant difference was found with Friedman test among the six illumination methods in depth perception, *χ*^*2*^(5) = 17.776, *p* = .003. The post-hoc Wilcoxon tests with a Bonferroni correction found that IML (*Z* = 0.0, *p* < .025), DIL (*Z* = 0.0, *p* < .025), SAL (*Z* = 0.0, *p* < .025), and PEL (*Z* = 2.0, *p* < .025) showed more clear depth information to participants than CVL. The results show that topology-aware illumination approaches helped participants more easily percept depth information of structures as we expected (H1). There were no other significant differences found between illumination methods on depth information.

For shape perception, a statistically significant difference was found among the six illumination methods, *χ*^*2*^(5) = 15.849, *p* = .007. The post-hoc Wilcoxon tests found that CSL (*Z* = 0.0, *p* < .025), IML (*Z* = 2.0, *p* < .025), SAL (*Z* = 0.0, *p* < .025), and PEL (*Z* = 1.5, *p* < .025) showed more clear shape information than CVL. The results indicate that topology-aware illumination approaches except topological distance based illumination helped participants better understand shape information of structures as we hypothesized (H2). Constant illumination also showed better shape information to participants than conventional approach. It was also found that PEL showed more clear shape information to participants than DIL illumination (*Z* = 0.0, *p* < .025). This result suggests that perceptual illumination was more effective in shape depiction than distance illumination.

For structural differences, there was a statistically significant difference found among the six illumination methods in structural difference perception, *χ*^2^(5) = 20.959, *p* < .001. The post-hoc Wilcoxon tests found that DIL (*Z* = 0.0, *p* < .025) and PEL (*Z* = 0.0, *p* < .025) showed more clear structural difference to participants than CVL. The results show that topology-aware illumination approach of distance illumination and perceptual illumination helped participants get more clear structural difference information as we expected (H3). It was also found that SAL (*Z* = 0.0, *p* < .025) and PEL (*Z* = 0.0, *p* < .025) showed more clear structural difference to participants than CSL. This result confirms the effectiveness of topology-aware illumination in depicting structural differences. Participants also found that PEL showed more clear structural difference than DIL (*Z* = 0.0, *p* < .025). The result suggests that perceptual illumination was more effective in depicting structural difference perception than distance illumination.

## Discussion

Previous illuminance design methods such as structure-aware approach [4] do not consider topological relations between structures at all and cannot depict variations of different structures effectively. Such approaches also cannot depict importance and topological differences of structures from the illumination perspective. Despite topology-aware transfer function approaches being developed [14, 15], they do not consider topology in illumination design to differentiate objects from the illumination perspective.

Compared with the previous work, we can see that our topology-aware illumination approach can generate illuminations and depict structural differences from the illumination perspective effectively. Because topology-aware illumination approach can define different illuminations for structures based on topological features, it shows more clear depth, shape, and structural difference information. Compared with conventional illumination approaches, the topology-aware illumination has following advantages: 1) It generates different illumination parameters for different sub-regions automatically. Users do not need to do trial-and-error interactions and only a simple slide bar is used as an interaction interface for specifying illumination speed or weights in the illumination models. Therefore, the exploration on a data can be got from the illumination perspective by the controlling of lighting speed or weights meaningfully instead of complicated adjustment of computer graphics concept on illumination. 2) The generated illuminations can depict importance of structures and structural relationships from the illumination perspective meaningfully and automatically based on topological features of volumetric data. 3) It optimizes illumination perception differences between structures based on the psychological theory, which allows users perceive structural difference effectively from the illumination perspective. 4) More interestingly, topology-aware illumination attenuation can dim out structures differently based on their topological importance in lighting attenuation, where less important structures are dimmed out while more important structures are still kept in lighting attenuation. 5) The proposed illumination approach also provides initial meaningful estimation of illumination parameters for their further fine tuning step. 6) Our approach does not require much user involvement, and it is even easy to use and understand for users who may not have much knowledge in illumination and visualization.

The effectiveness of our method is mainly affected by the quality of the contour tree, which is originally related to the quality of volumetric data set such as signal-to-noise ratio (SNR). By preprocessing original volumetric data to filtering noise, more precise contour tree can be obtained and thus our approach becomes more effective in illumination designs. The paper used the classical Blinn-Phong model as an example to demonstrate the advantages of our approach in illumination design. The proposed approach can also be adapted to any other illumination models.

## Conclusions

This paper introduced a new paradigm for illumination design in volume rendering based on data topology. In the proposed framework, Stokes’ law of sound attenuation was adapted to model the lighting attenuation based on topological attributes. Topological distance and topological saliency were also integrated into the illumination design through a lighting transfer function mechanism to emphasize structures. Furthermore, a two-phase topology-aware illumination perception contrast model was proposed to maximize illumination perception differences between structures based on the psychological concept of Just-Noticeable-Difference. This two-phase topology-aware illumination perception contrast model allowed more efficient and meaningful automation of illumination generations, and the exploration on the data can be conducted from the illumination perspective by the controlling of weighting factors meaningfully instead of complicated adjustment of computer graphics concept on illumination. Experiments showed that our approach was more effective in depth and shape depiction, as well as providing higher perceptual differences between structures.

In the future, we will investigate topology-aware illumination in more general illumination models besides the classical Blinn-Phong model for more complex illumination designs. We also plan to investigate other perceptual psychological models in illumination to enhance perceptual differences of structures in volume rendering.

## Abbreviations

CSL: Constant diffuse
CT: Contour tree
CVL: Conventional illumination
DIL: Distance illumination
IML: Importance illumination
JND: Just-Noticeable-Difference
MS: Morse-Smale
PEL: Perceptual illumination
SAL: Saliency illumination
SNR: Signal-to-noise ratio
